# Life-threatening rupture of an external iliac artery pseudoaneurysm caused by necrotizing fasciitis following laparoscopic radical cystectomy: a case report

**DOI:** 10.1186/1756-0500-7-290

**Published:** 2014-05-10

**Authors:** Shinro Hata, Ryuta Satoh, Toshitaka Shin, Kenichi Mori, Yasuhiro Sumino, Fuminori Satoh, Hiromitsu Mimata

**Affiliations:** 1 Department of Urology, Faculty of medicine, Oita University, Idaigaoka 1-1, Hasama-cho, Yufu, Oita Prefecture 879-5593, Japan

**Keywords:** Bladder cancer, Pseudoaneurysm, Laparoscopic, Pelvic lymphadenectomy

## Abstract

**Background:**

Pseudoaneurysms are caused by trauma, tumors, infections, vasculitis, atherosclerosis and iatrogenic complications. In this paper, we report about a patient with rupture of an external iliac artery pseudoaneurysm, which lead to hemorrhagic shock, after undergoing laparoscopic radical cystectomy and extended pelvic lymphadenectomy.

**Case presentation:**

The patient was a 68-year-old Japanese male diagnosed with invasive bladder cancer. Laparoscopic radical cystectomy and extended pelvic lymphadenectomy were performed. On postoperative day 12, he developed a high fever and an acute inflammatory response with redness and swelling in the right inguinal region. He was diagnosed with necrotizing fasciitis and underwent debridement. On postoperative day 42, a sudden hemorrhage developed from the open wound in the right inguinal region. He was diagnosed with external iliac artery pseudoaneurysm rupture by computed tomography.

**Conclusion:**

These complications occur extremely rarely after cystectomy with pelvic lymphadenectomy. There are no reports to date on these complications following laparoscopic cystectomy with pelvic lymphadenectomy.

## Background

It is extremely rare for formation and rupture of an iliac artery pseudoaneurysm after pelvic surgery. Usually, pseudoaneurysms are caused by trauma, tumors, infections, vasculitis, atherosclerosis and iatrogenic complications [1]. In this paper, we report about a patient with rupture of an external iliac artery pseudoaneurysm, which lead to hemorrhagic shock, after undergoing laparoscopic radical cystectomy, extended pelvic lymphadenectomy and ileal conduit diversion.

## Case presentation

**Figure 1:**
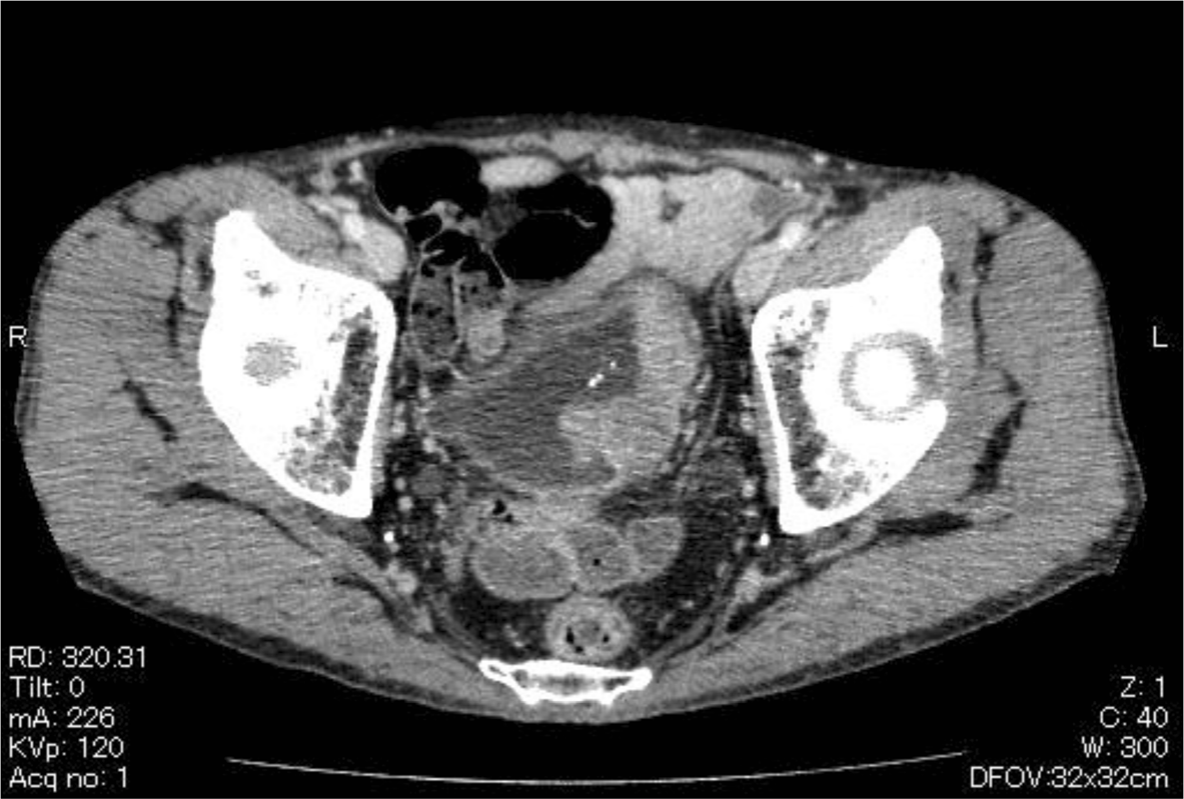
Preoperative CT findings of the pelvis; CT showed the invasive bladder cancer (clinical stage T3a) and the normal external iliac artery.

**Figure 2:**
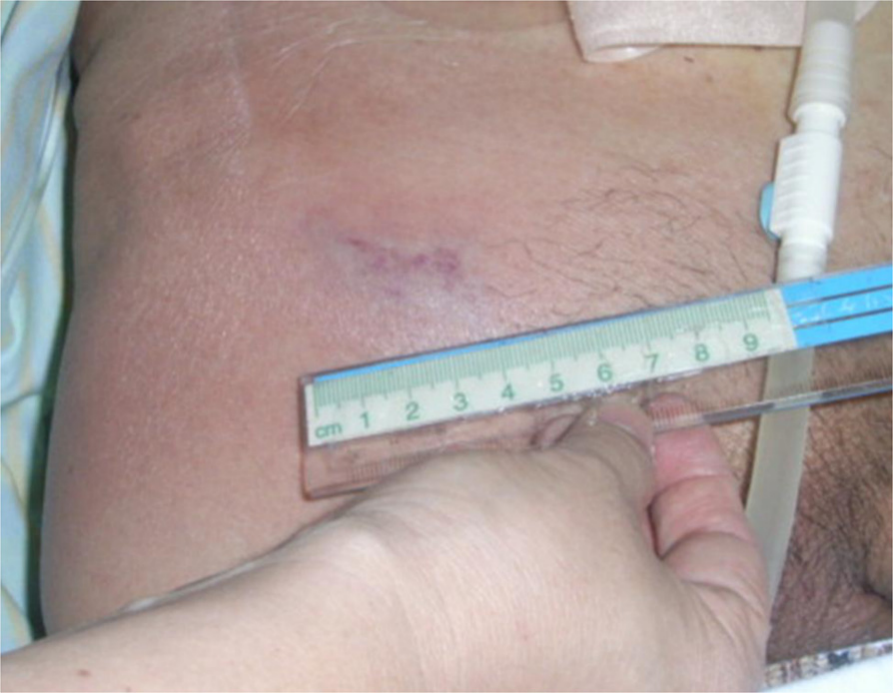
Appearance of right inguinal region; redness and swelling were recognized.

**Figure 3:**
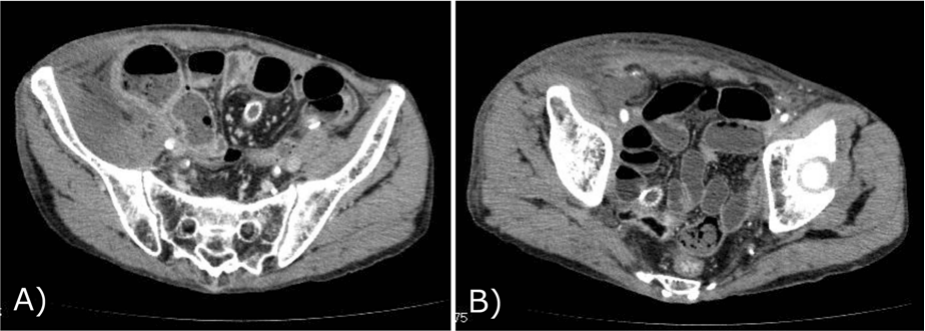
**CT findings before the debridement; A) showed the retroperitoneal abscess. B)** showed the normal external iliac artery.

**Figure 4:**
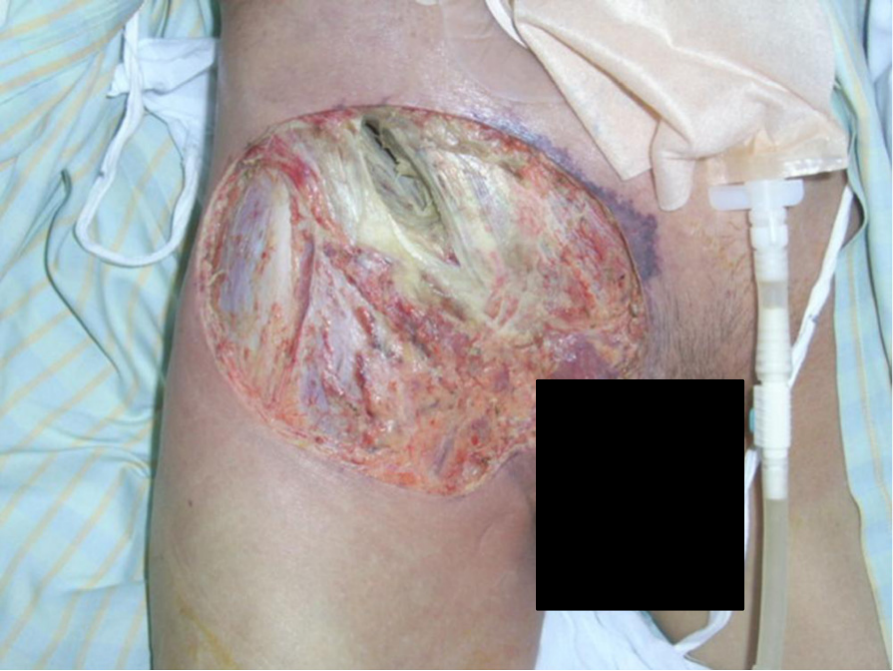
Postoperative appearance; debridement for retroperitoneal abscess.

**Figure 5:**
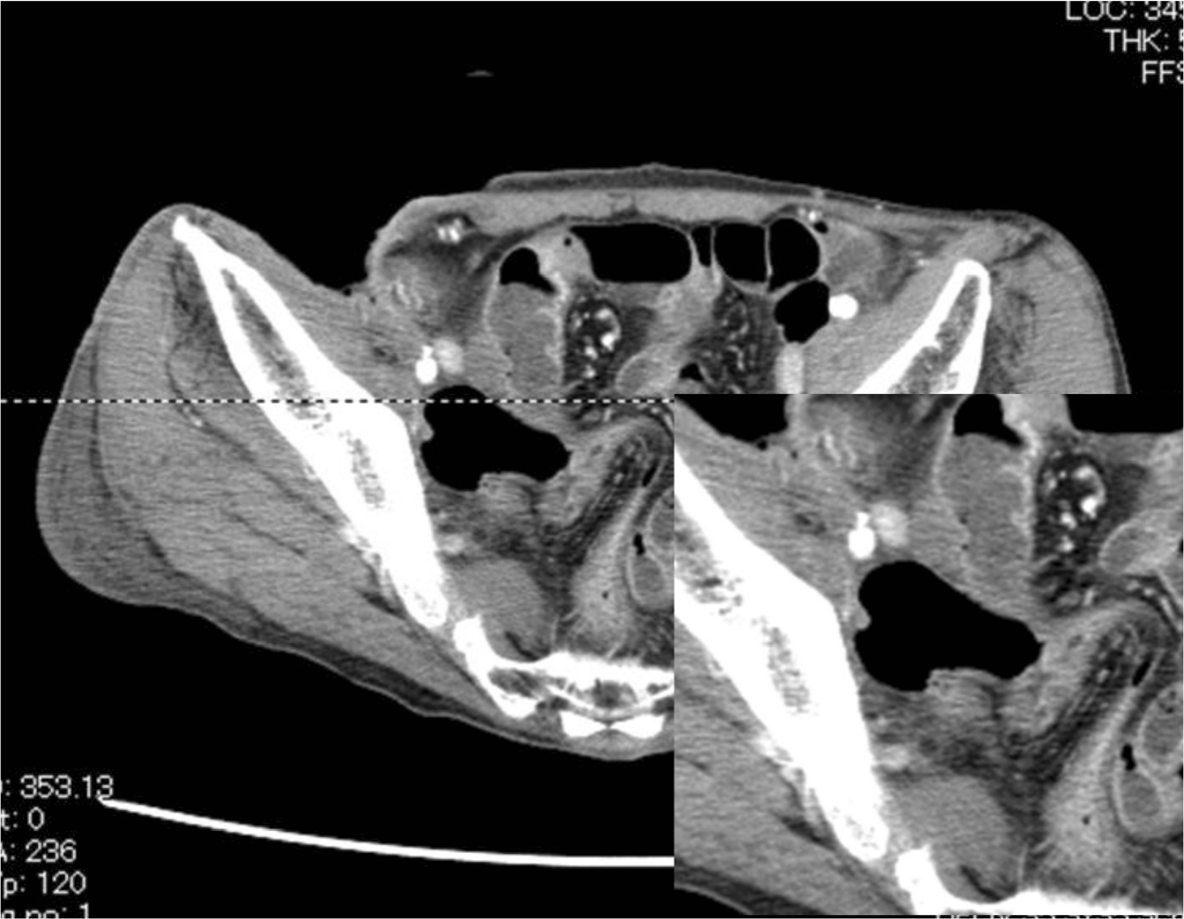
CT findings of the pelvis; CT showed the pseudoaneurysm of external iliac artery.

**Figure 6:**
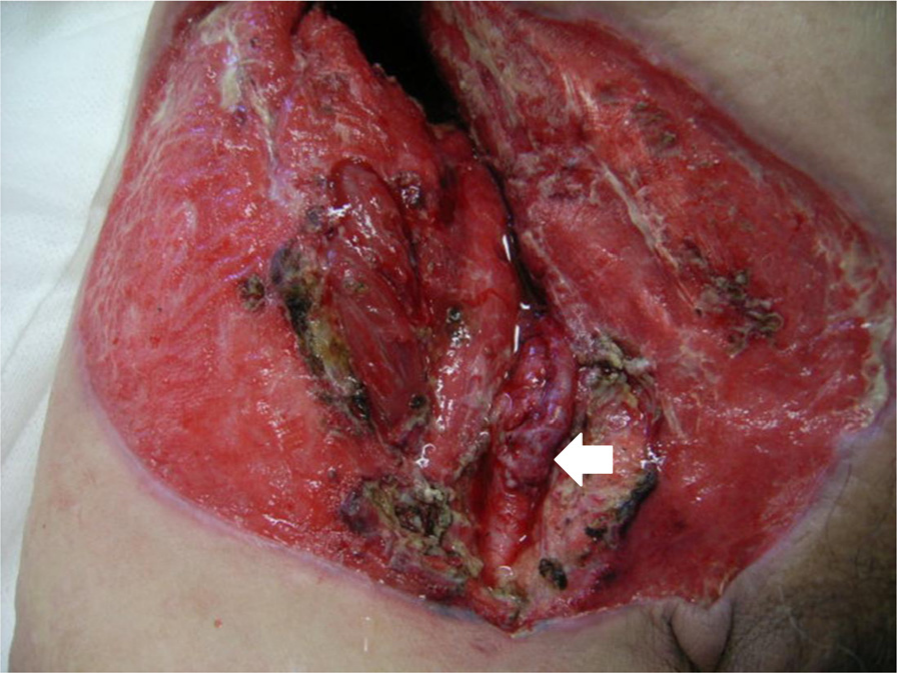
Postoperative appearance (bypass surgery); the arrow showed the great saphenous vein.

The patient was a 68-year-old Japanese male diagnosed with invasive bladder cancer with clinical stage T3a N0 M0 (Figure 1). His body mass index (BMI) was 17.4 kg/m^2^ and he had a history of alcoholic hepatitis. Liver function before the surgery was good. Laparoscopic radical cystectomy, extended pelvic lymphadenectomy and ileal conduit diversion were performed. No intraoperative complications were observed, the surgery time was 679minutes, and the estimated blood loss was 340 ml. Pathological stage was pT3a pN0. Although the early postoperative course was favorable, the patient developed a high fever and an acute inflammatory response with redness and swelling in the right inguinal region on postoperative day (POD) 12 (Figure 2). Intravenous antibiotics were initiated but skin necrosis ensued. The patient was diagnosed with necrotizing fasciitis and retroperitoneal abscess by computed tomography (CT) (Figure 3). Then the patient underwent debridement on POD 13 (Figure 4). At this time the external iliac artery could not be identified. After debridement, even though the local inflammatory findings were improved, the levels of C-reactive protein (CRP) remained elevated (3–4 mg/dl). Six weeks after initial surgery, there was noted to be excessive spontaneous bleeding from the right inguinal wound, resulting in hemorrhagic shock. The patient was diagnosed with external iliac artery pseudoaneurysm rupture by CT (Figure 5). Bypass graft surgery was performed using the great saphenous vein (Figure 6). *Methicillin-resistant Staphylococcus aureus* (*MRSA*) was cultured from the wound, and the patient’s condition slowly improved on intravenous daptomycin. A pedunculated femoral flap and split-thickness skin graft were performed to cover the inguinal wound, and the patient was discharged home 12weeks after initial surgery. At twelve months follow up, there was no evidence of aneurysm recurrence and no prolonged limb deficit.

### Discussion

Pseudoaneurysms are a result of damage to the vascular wall due to factors such as trauma, tumor, infection, vasculitis, atherosclerosis, or iatrogenic injury [1]. The iliac artery is a rare site of the onset, accounting for 3 to 18% of all cases. Pelvic surgery rarely results in formation or rupture of iliac artery pseudoaneurysms, and the frequency is unknown [2,3]. Simon *et al*. have only reported one case of pseudoaneurysm formation in the common iliac artery associated with open radical cystectomy [4]. Vascular injury is rare in laparoscopic cystectomy, with a rate of 0 to 3.7% [5-7].

Rapid revascularization is usually required since pseudoaneurysm rupture causes shock due to heavy bleeding. Endovascular interventions include surgical revascularization (vascular grafts and autologous vessels), and more recently, stent grafts. Ricciardi *et al.* reported a case of external iliac artery pseudoaneurysm rupture following pelvic lymphadenectomy for cervical cancer [2]. They performed endovascular intervention using a covered stent, and advocate their use as first-line treatment in these kinds of cases, as it is possible to achieve faster control of bleeding than with surgical techniques.

The present case was an infectious pseudoaneurysm that spread from a retroperitoneal abscess, and thus foreign objects should not be left within the body. At the time of debridement, *Staphylococcus epidermides* was detected in a subcutaneous pus swab and *MRSA* was detected from a retroperitoneal pus swab. Therefore, we conducted revascularization using an autologous graft from the great saphenous vein without a covered stent or vascular graft.

In this case, the factors leading to pseudoaneurysm rupture were thought to be the spread of infection from the retroperitoneal abscess and the weakening of the vascular wall associated with the lymphadenectomy. The factors that triggered the formation of the retroperitoneal abscess are unclear. We suggest that the cause of pseudoaneurysm formation was microscopic injury of the external iliac artery which was not detected during surgery. Furthermore, the vascular wall became brittle due to *MRSA* infection, ultimately leading to rupture.

## Conclusions

External iliac artery pseudoaneurysms are rare complications of laparoscopic radical cystectomy with pelvic lymphadenectomy. When recognized and treated promptly, good long-term functional outcomes can be achieved.

## Consent

Written informed consent was obtained from the patient for publication of this case report and any accompanying images. A copy of the written consent is available for review by the Editor-in-Chief of this journal.

## Abbreviations

POD: Postoperative days; CT: Computed tomography; BMI: Body mass index; MRSA: Methicillin-resistant Staphylococcus aureus.

## Competing interests

The authors declare that they have no competing interests.

## Authors’ contributions

SH performed the majority of this study and drafted the manuscript. RS and TS surveyed the literature. KM and HM critically revised the manuscript. YS, FS, and HM participated in the design and interpretation of this study under supervision. All authors read and approved the final manuscript.
